# Editorial: Identification of novel biomarkers for pancreatic and hepatocellular cancers

**DOI:** 10.3389/fonc.2022.1056002

**Published:** 2022-11-08

**Authors:** Laura Matteucci, Ilario Giovanni Rapposelli, Alessandro Passardi

**Affiliations:** Department of Medical Oncology, IRCCS Istituto Romagnolo per lo Studio dei Tumori (IRST) “Dino Amadori”, Meldola, Italy

**Keywords:** pancreatic cancer, hepatocellular carcinoma, biomarkers, prognosis, predictive, translational oncology

Pancreatic and hepatocellular cancers are among the most aggressive human malignancies and a major cause of cancer mortality in the world (1). Although groups at high risk for these malignancies have been recognized, screening and early detection strategies have not been successful yet. For both tumors, diagnosis often comes at advanced stage, and systemic therapy is the only treatment option. Unfortunately, systemic treatments such as chemotherapy, targeted therapies and immunotherapy often have limited clinical benefit. Hopefully, our evolving understanding of the disease biology and the advancements of molecular biology will provide new approaches for early detection and tailored therapy. In particular there is an increasing interest in the identification of potential novel diagnostic, prognostic and/or predictive biomarkers in the field of pancreatic and hepatocellular cancers, with the aim to improve patient prognosis and overall survival rates.

*Pancreatic cancer* (PC) has the highest mortality rate of all major cancers and it is currently the third leading cause of cancer-related death after lung and colon cancers (1). Even in the resectable setting, high rates of recurrences confer a dismal prognosis. Therefore, in the last years there has been an increasing trend toward neoadjuvant chemotherapy (NAC) for resectable and borderline resectable disease, in order to control possible micrometastases and select patients with potential benefit from radical resection (Oba et al.). However, a subset of patients does not benefit from NAC, and the optimal treatment schedule is not yet defined. Thus predictive markers are awaited to identify patients who may benefit from NAC. In this context, a proteomic analysis was performed by Sahni et al. from tissue samples of PC patients treated with NAC: GRP78, CADM1, PGES2, and RUXF were shown to be the best predictors of poor response to treatment. Notably, pathway analysis indicated an activation of immune response pathways in good responders, highlighting the fundamental role of cross-talk of PC cells and immune microenvironment (2).

The evaluation of diagnostic and prognostic markers to better identify and stratify patients with PC is another important field of research, and in the present special issue some preclinical trials have been presented. Wang et al. explored the value of serum biomarkers in the differential diagnosis between serous and mucinous pancreatic cystic neoplasms. They showed that ‘lymphocyte × ALB’ decrease and CA19.9 increase had good differential diagnostic efficacy, being relevant risk factors for mucinous cystic neoplasms. Kong et al. developed a prognostic prediction model based on four CpG sites using the Cancer Genome Atlas (TCGA) and the International Cancer Genome Consortium (ICGC) datasets as discovery and validation cohorts, respectively. They identified DNAJB1, a suppressor of p53-mediated apoptosis (3), as a potential diagnostic and prognostic biomarker for PC. Next, a nomogram model based on the independent prognostic factors was constructed, leading to a new method for predicting the prognosis of patients with PC. The protein hypoxia-inducible lipid droplet-associated (HILPDA) expression was analyzed in pan-cancer data from The Cancer Genome Atlas (TCGA) database. It was shown to be a marker of poor prognosis **(** Liu et al. **)**. Interestingly, Kong et al. reported high immune infiltration in the low-risk group, while Liu et al. showed the association of the poor prognosis marker HILPDA with tumor-associated macrophage infiltration and the expression of immunosuppressive factors, underscoring once again the importance of the interplay with immune system for PC progression (4). A panel of four serum biomarkers (S100A2, S100A4, Ca-125 and Ca 19-9) was tested in PC patients and healthy controls, providing the potential, to be validated in larger cohorts, to diagnose and stratify PC patients based on their prognostic outcomes (Mehta et al.). This study pursues the promising strategy of early detection of PC by liquid biopsy (5). Chen et al. provided an interesting review on the impact of platelets on PC, including the molecular mechanisms of cancer onset, fibrosis and thrombosis, immune escape, as well as drug resistance mechanisms and targeted therapy (Chen et al.).

*Hepatocellular carcinoma* (HCC) accounts for about 85%-90% of all primary liver cancers and is the fourth cause of cancer-related mortality worldwide (1). Most cases of HCC occur in patients with chronic liver disease and may present with non-specific symptoms such as jaundice, abdominal pain, nausea and vomiting, or fatigue. The prognosis of HCC is affected by tumor stage and liver function, therefore early diagnosis and correct management of liver disease are crucial. However, the diagnosis of HCC, in particular those cases with negative alpha-fetoprotein, is often challenging, and new diagnostic biomarkers are awaited (Wang et al.). To this end, it is essential to boost studies aimed at defining the molecular mechanisms underlying the development of HCC. Luet al. carried out a preclinical study on the role of FAM21C in promoting malignant progression of HCC both *in vitro* and *in vivo*. FAM21C led to the inhibition of the capping protein CAPZA1, which drives F-actin cytoskeleton remodelling, and thus promoted invasion and migration of HCC cells. Xu et al. carried out an interesting review on the miRNAs that are most commonly up-regulated or downregulated in liver tumor tissues and plasma/serum of hepatitis B virus (HBV)-related HCC patients. Indeed, patterns of miRNA expression in HCC differ according to HCC aetiology (e.g., viral, alcoholic liver disease, nonalcoholic steatohepatitis) (6). Indeed, miRNAs, isolated from serum or from extracellular vesicles, have a promising role both as early detection biomarkers and as therapeutic targets (7, 8). Liu et al. explored the role of the RNA binding protein Zinc Finger CCHC-Type Containing 17 (ZCCHC17) in the diagnosis and prognosis of HCC. They found that protein and mRNA levels of ZCCHC17 were significantly higher in tumor than in normal tissues, moreover HCC patients with high ZCCHC17 expression had a worse prognosis. The authors also highlighted a possible role of the protein in the regulation of immune cells in the tumor microenvironment, suggesting future applications in the immunotherapy of HCC.

Surgical resection or ablation of the tumor are the treatment of choice for HCC, but only 5–15% of patients are suitable for surgical resection due to the extent of disease or poor liver function, moreover among operated patients long-term survival rates remain unsatisfactory because of high recurrence rates (9). Presence of microvascular invasion (MVI) is considered one of the most important risk factors related to tumor recurrence (10). For this reason non-invasive preoperative prediction of MVI might be vital for precise surgical decision-making and patient prognosis definition: the identification of such predictive model by means of a machine learning algorithm (Liu et al.) gives an outlook of the potential impact of artificial intelligence in the management of HCC (11). In order to further refine the management of resectable disease, Fan et al. performed a meta-analysis of 11 studies with 7,442 HCC patients undergoing hepatectomy, and found that a low preoperative serum prealbumin level was significantly associated with poor overall and recurrence-free survival.

In the disease which has spread beyond the liver, treatment with tyrosine kinase inhibitors (TKIs) or immune checkpoint inhibitors (ICIs) is recommended (12–14). Despite the increasing number of therapeutic options, predictive markers of efficacy are still lacking. Zhao et al. evaluated the role of sarcopenia and systemic inflammation response index (SIRI) with encouraging results. Interestingly, sarcopenia and high SIRI were associated with reduced survival in HCC patients treated with TKIs and ICIs. Also, they show that sarcopenia may affect inflammatory states and immune microenvironment, a crucial effect if considering the increasing immunotherapeutic options in HCC (15).

However, there is great interest in expanding our knowledge about potentially druggable alterations in HCC (Niu et al.). HER2 aberrations have been observed in about 14.9% of advanced biliary tract tumors (Kim et al.) and are present in even lower percentages in HCC. With the aim to identify new potential targets, Elmas et al. performed a proteomics analysis on 260 HBV-related HCC and identified some overexpressed targets which deserve further studies such as PDGFRB, FGFR4, ERBB2/3, CDK6 kinases and MFAP5, HMCN1, and Hsp. Furthermore, the expression of FGFR4 and Hsp were significantly associated with response to their inhibitors. Glypican-3 (GPC3) has been recently studied as a potential marker of diagnosis and prognosis of HCC, as well as a potential target for targeted treatments, as reviewed by Zheng et al. Lysyl oxidase (LOX) and copper metabolism MURR1 domain (COMMD) family members are being developed with the same potential (Sun et al., Fang et al.). A different, interesting approach for targeted therapy of HCC exploits FDG PET/CT: indeed, metabolism-associated gene signatures showed prognostic value, and a potential utility for the identification of appropriate patients for metabolism-targeted therapy (Lee et al.).

## Author contributions

AP wrote the first draft of the manuscript, LM and IGR contributed to manuscript revision, read, and approved the submitted version. 

## Conflict of interest

The authors declare that the research was conducted in the absence of any commercial or financial relationships that could be construed as a potential conflict of interest.

## Publisher’s note

All claims expressed in this article are solely those of the authors and do not necessarily represent those of their affiliated organizations, or those of the publisher, the editors and the reviewers. Any product that may be evaluated in this article, or claim that may be made by its manufacturer, is not guaranteed or endorsed by the publisher.
